# Navigating through the Lipid Metabolism Maze: Diagnosis and Prognosis Metabolites of Hepatocellular Carcinoma versus Compensated Cirrhosis

**DOI:** 10.3390/jcm11051292

**Published:** 2022-02-26

**Authors:** Iuliana Nenu, Horia Stefanescu, Bogdan Procopet, Zeno Sparchez, Iulia Minciuna, Tudor Mocan, Daniel Leucuta, Corina Morar, Mircea Grigorescu, Gabriela Adriana Filip, Carmen Socaciu

**Affiliations:** 1 3rd Medical Department, “Iuliu Hatieganu” University of Medicine and Pharmacy, 400162 Cluj-Napoca, Romania; procopet.bogdan@umfcluj.ro (B.P.); sparchez.zeno@umfcluj.ro (Z.S.); minciuna.iulia@elearn.umfcluj.ro (I.M.); mocan.tudor@umfcluj.ro (T.M.); grigorescu.mircea@umfcluj.ro (M.G.); 2 Regional Institute of Gastroenterology and Hepatology, 400162 Cluj-Napoca, Romania; 3 Department of Medical Statistics, “Iuliu Hatieganu” University of Medicine and Pharmacy, 400349 Cluj-Napoca, Romania; leucuta.daniel@umfcluj.ro; 4 Center for Applied Biotechnology BIODIATECH, SC Proplanta, 400478 Cluj-Napoca, Romania; corina.hebristean@yahoo.co.uk (C.M.); csocaciudac@gmail.com (C.S.); 5 Department of Physiology, “Iuliu Hatieganu” University of Medicine and Pharmacy, 400006 Cluj-Napoca, Romania; gabriela.filip@umfcluj.ro

**Keywords:** hepatocellular carcinoma, cirrhosis, lipid metabolites, biomarkers

## Abstract

(1) Background: The pursuit of finding biomarkers for the diagnosis and prognosis of hepatocellular carcinoma (HCC) has never been so paramount in the days of personalized medicine. The main objective of our study is to identify new biomarkers for diagnosing HCC, and to identify which patients are at risk of developing tumor recurrence, decompensation, or even possesses the risk of cancer-related death. (2) Methods: We have conducted an untargeted metabolomics study from the serum of 69 European patients—32 compensated cirrhotic patients without HCC (controls), and 37 cirrhotic patients with HCC with compensated underlying liver disease (cases), that underwent curative treatment (surgery or ablation), performing ultra-high-performance liquid chromatography coupled with electrospray ionization quadrupole time-of-flight mass spectrometry (UHPLC-QTOF- (ESI+)-MS) with an emphasis on lipid metabolites. (3) Results: 1,25-dihydroxy cholesterol (m/z = 419.281), myristyl palmitate (m/z = 453.165), 25-hydroxy vitamin D2 (m/z = 413.265), 12-ketodeoxycholic acid (m/z = 391.283), lysoPC (21:4) (m/z = 558.291), and lysoPE (22:2) (m/z = 534.286) represent notable biomarkers that differentiate compensated cirrhosis from early HCC, and ceramide species are depleted in the serum of HCC patients. Regarding prognosis, no metabolite identified in our study could determine tumor relapse. To distinguish between the HCC patients that survived curative treatment and those at risk that developed tumor burden, we have identified two notable phosphocholines (PC (30:2); PC (30:1)) with AUROCs of 0.820 and 0.807, respectively, that seem to increase when patients are at risk. In a univariate analysis, arachidonic acid was the only metabolite to predict decompensation (OR = 0.1, 95% CI: 0–0.16, *p* < 0.005), while in the multivariate analysis, dismally, no variable was associated with decompensation. Furthermore, in the multivariate analysis, we have found out for the first time that the increased expression of 1,25-dihydroxy cholesterol, myristyl palmitate, 12-keto deoxycholic acid, lysoPC (21:4), and lysoPE (22:2) are independent markers of survival. (4) Conclusions: Our study reveals that lipids play a crucial role in discriminating compensated cirrhosis and early hepatocellular carcinoma, and might represent markers of survival and prognosis in personalized and minimally invasive medicine.

## 1. Introduction

One of the challenges that hepatologists face nowadays is the troublesome occurrence of hepatocellular carcinoma (HCC), the sixth most common cause of cancer worldwide, with an increasing incidence. Moreover, the World Health Organization estimates that more than a million people will die from liver cancer in 2030 [1,2]. In addition, due to late or limiting diagnosis methods, HCC has become more and more frequent on the doctor’s agenda, often diagnosed in advanced stages, with a median overall survival time of 6–8 months [3].

Although α-fetoprotein (AFP) is the most often used screening biomarker in clinical practice for early HCC detection, predicting tumor recurrence, and monitoring the oncological treatment, its poor sensitivity and specificity make it an unsatisfactory marker [4]. For this reason, new biomarkers are needed to identify among cirrhotic patients those at risk of developing liver malignancy, or to monitor treatment efficacy in the case of oncological patients. Along these lines, the concept of liquid biopsy has appeared in oncology for a minimally invasive positive diagnosis, prognosis, and overall disease monitoring. The term liquid biopsy can apply to cancer by-products, including circulating tumor cells (CTC), cell-free DNA (cfDNA), cell-free RNA (cfRNA), microRNA (miRNA), and extracellular vesicles (EVs), and last but not least, to metabolomics as well [5]. Because no organ other than the liver encompasses a myriad of lipids and other metabolites that are constantly interchanging in many biochemical reactions, metabolomics, which represents a compressive fingerprint of cell metabolism, also plays a crucial role in pursuing new diagnostic biomarkers and in identifying biochemical networks involved in prognosis and treatment monitoring [6,7]. 

Metabolic reprogramming is a hallmark characteristic of all cancer cells [8]. The most well-studied metabolic peculiarity is the Warburg effect, in which cancer cells use aerobic glycolysis instead of mitochondrial oxidative phosphorylation, leading to increased lactate production with a consequent role in cellular proliferation [9,10]. Because the liver is the primary metabolic hub of lipid metabolism through lipid homeostasis and energy balance maintenance, it is plausible to assume that many biochemical pathways involving lipids are disrupted [11]. Indeed, increased lipogenesis and lipolysis promote cancer cell growth, proliferation, and survival [11]. Moreover, fatty acid metabolism is enhanced, and phosphatidyl-choline (PC) and lyso-phosphatidylcholine (LPC) increase, while phosphatidyl-ethanolamine, -serine (PS) and -inositol, and ceramides are markedly reduced in HCC [11,12]. Other lipid metabolites play a double-edged sword role. Sphingolipid metabolism is quite controversial because ceramides induce a proapoptotic effect, whereas their phosphorylated compounds are involved in cancer growth, survival, and motility [13]. There are limited data regarding the involvement of ceramides and sphingosine-1 phosphate (S1P) as significant regulators of hepatocarcinogenesis, and their possible roles as biomarkers in HCC [14]. Some authors report a highly significant upregulation of long and very-long-chain ceramides (C16–C24) in the serum of patients with HCC compared to patients with cirrhosis, while other authors find a ceramide depletion in the serum of HCC patients [15,16]. Bile acids also have a dual role, with both protective and pathogenic roles being a matter of debate in drug-induced liver injury, NAFLD, and colon and liver cancers [17].

The main objective of our study is to identify new serum biomarkers, mainly lipids, and to define prognosis markers of early hepatocellular carcinoma, based on ultra-high-performance liquid chromatography coupled with electrospray ionization quadrupole time-of-flight mass spectrometry (UHPLC-QTOF- (ESI+)-MS). The present study identifies which patients are at risk of developing tumor recurrence, decompensation, and increased mortality. 

## 2. Patients and Methods

### 2.1. Patient Characteristics and Sample Collection

We have conducted a retrospective analysis of a prospectively collected database. The patients were admitted during 2016–2017 at the “Prof. Octavian Fodor” Regional Institute of Gastroenterology and Hepatology Cluj-Napoca, Romania, as follows: 37 patients with HCC (BCLC stages 0, A, and B) and 32 with compensated cirrhosis were admitted, with a period of three years of follow-up. The diagnosis of HCC was established based on the EASL guidelines for HCC using imaging and/or histopathology, and the stadialisation was performed according to the Barcelona clinic liver cancer (BCLC) score. All patients with compensated cirrhosis, representing the control group, had hepatitis C virus etiology and were referred to interferon-free treatment. The diagnosis of liver cirrhosis was made using non-invasive methods such as abdominal ultrasound, transient elastography, serology, and liver biopsy in certain situations. HCC confirmation included two dynamic imaging examinations according to the EASL guidelines. Clinical characteristics included age, gender, and the etiology of the underlying liver disease. Liver function was determined using the Child-Pugh score, the model for end-stage liver disease (MELD) score, the alpha-fetoprotein (AFP), aspartate aminotransferase (AST), and alanine aminotransferase (ALT) levels, the platelet count, and the gamma-glutamyl transpeptidase (GGT) level. 

Hepatic venous pressure gradient (HVPG) was performed to determine the portal hypertension grade, where clinically significant portal hypertension was defined over 10 mmHg [18]. Tumor size and tumor number were assessed via imagistic tools at the time of inclusion, and, for HCC patients, the BCLC stage was determined. Contrast-enhanced CT or contrast-enhanced MRI were performed every three months during the first year and at least every 4–6 months thereafter to monitor disease progression. Independent physicians performed all follow-up examinations without prior knowledge of the study. The development of decompensation events was noted in the HCC group and was defined by the appearance of jaundice, ascites, hepatorenal syndrome, hepatic encephalopathy, and/or variceal bleeding. In the control group, patients with liver nodules or those with a history of decompensation or current symptoms of such were excluded. Patients with HCC developed on the non-cirrhotic liver, patients with advanced HCC (BCLC classes C and D), and those with concomitant cancer other than HCC were also excluded from the study group. Pregnant patients, as well as patients younger than 18 years old were excluded. The patients were followed up until November 2020. The study was performed according to the Declaration of Helsinki, and both groups signed the informed consent of the examination approved by the local ethical guidelines for storing biological samples. The cohort was prospectively studied, and serum lipid parameters were analyzed in retained serum samples, stored previously at −80 °C.

### 2.2. Metabolomic Analysis

#### 2.2.1. Sample Preparation

The patients included in this study had blood collected by venipuncture in sterile vacutainers anticoagulant, and samples were kept at −80 °C. They were labeled using confidential numerical codes. A volume of 0.6 mL methanol (99%) was added for each volume of 0.2 mL of plasma, and the mixture was vortexed to precipitate proteins for 30 s. Afterward, the composition was kept for 5 min in an ultrasonication instrument, followed by 5 min at −20 °C. The supernatant was collected following centrifugation at 10,000 rpm for 10 min (4 °C).

Afterward, probes were subjected to ultrasound in a water bath for 5 min and left at −20 °C to increase protein precipitation. The samples were centrifuged at 5000 rpm for 10 min to separate the protein supernatant. The supernatant was collected and filtered using nylon filters (0.2 μm). The samples were placed in vials, and then in an autosampler to be injected into the UHPLC MS system.

#### 2.2.2. UHPLC-QTOF-ESI+-MS Analysis

Plasma metabolomic profiling was performed using ultra-high-performance liquid chromatography coupled with electrospray ionization-quadrupole-time of flight-mass spectrometry (UHPLC-QTOF-ESI+-MS) in a ThermoFisher Scientific UHPLC Ultimate 3000 instrument equipped with a quaternary pump, a Dionex delivery system, and MS detection equipment with MaXis Impact (Bruker Daltonics). The metabolites were separated using a Thermo Scientific Acclaim C18 column (3 μm, 2.1 × 50 mm, pore size 30 nm) at 40 °C. The mobile phase consisted of 0.1% formic acid in water (A) and 0.1% formic acid in acetonitrile (B). The flow rate was set at 0.5 mL min^−1^. The gradient was: 5 to 15% A (0–3 min), 15–50% A (3–6 min), 50–95% (6–9 min), isocratic until 15 min, and afterwards decreased from 95 to 5% (15–20 min). The elution time was set for 20 min. The volume of the injected extract was 5 μL, the column temperature was 40 °C. Several QC samples obtained from each group were used in parallel to calibrate the separations.

Metabolite identification was performed using specific MS parameters: ionization mode ESI+ positive, MS calibration with sodium formate, capillary voltage 3500 V, pressure for the nebulizing gas 2.8 bar, drying gas flow 12 l/min, drying temperature 300 °C. The control of the instrument and the data processing were conducted using the specific software TofControl 3.2, HyStar 3.2, Data Analysis 4.2 (Bruker, Daltonics), and Chromeleon, respectively. 

### 2.3. Data Processing and Statistical Analysis

The base peak chromatograms and all MS spectra were first processed by Compass DataAnalysis 4.2 (Bruker Daltonics, GmbH, Bremen, Germany), using the find molecular feature (FMF) algorithm, and matrix generation was achieved using Profile Analysis 2.1 (Bruker Daltonics, GmbH, Bremen, Germany). The time alignment, spectral background extraction, MS recalibration, normalization by the sum of the bucket values in analysis, and an 80% bucket filter were the chosen parameters. 

MetaboAnalyst v5.0 online software was used for univariate and multivariate analyses. The matrices representing the peak intensity versus m/z values for each sample and subgroups of samples were tested using the most relevant statistical parameters to reflect the discrimination between groups, the prediction, and the correlation maps. Therefore, the comparative statistical approaches selected were the volcano test, the variable importance in the projection (VIP) values, the scores and loadings plots of principal component analysis (PCA) and partial least square discriminant analysis (PLSDA) including cross-validation parameters, the random forest-based prediction, and the calculation of *p*-values by *t*-test via ANOVA. The same software was applied to build the receiver operating characteristic (ROC) curves and area values under ROC curves (AUC) to evaluate the potential biomarkers’ sensibility and selectivity. Finally, enrichment analysis and pathway matches were established to find the significant metabolites for altered pathways. The relevant molecules, according to statistical analysis, were identified by searches on specialized databases, such as Lipid Maps (http://www.lipidmaps.org (accessed on 20 December 2021)) and the Human Metabolome Database (http://www.hmdb.ca (accessed on 20 December 2021)), and PubChem (https://pubchem.ncbi.nlm.nih.gov/ (accessed on 20 December 2021). The PubChem database codes of each identified metabolite were included.

### 2.4. Data Processing and Statistical Analysis for Prognosis

Regarding prognosis, the overall survival was defined as the time from treatment until death or the study ending date (November 2020). Disease-free survival was defined as the time from treatment until recurrence or the study’s end date (November 2020). Further, known predictors for survival were added as adjusting variables in multivariate Cox regression models. The proportional hazard assumption was checked with a formal statistical test for all models, while the linear functional form for continuous variables was checked using model residuals plots inspection. For multivariate models, multicollinearity was checked with variance inflation factors. The two-tailed *p*-value was computed for all statistical tests, and the results were statistically significant for values below 0.05. Data were analyzed using the R environment for statistical computing and graphics (R Foundation for Statistical Computing, Vienna, Austria), version 3.6.3 [R Core Team. R: A Language and Environment for Statistical Computing and IBM SPSS Statistics 25.0.] (IBM, Armonk, NY, USA).

## 3. Results

### 3.1. Patient Characteristics

The present study included patients with HCC (group HCC) and patients with compensated cirrhosis as controls (group C). Because all HCV patients from the control group received interferon-free treatment, they were rigorously evaluated clinically and biochemically, and imaging (ultrasound ± CT) was performed. In effect, the national protocol in 2016 banned interferon-free therapy in patients who had cancers, including HCC and decompensated (or a history of decompensated) liver disease. Thus, all of our control patients had compensated hepatitis C cirrhosis. Precisely for this reason, knowing with certainty that the patients from the control group did not have liver tumors, we compared patients with liver cirrhosis with those with HCC to determine different biomarkers of diagnosis and prognosis.

Most patients with HCC had compensated liver disease, with hepatitis C virus (HCV) representing the primary etiology—43.24%, followed by alcoholic cirrhosis (32.4%), and chronic hepatitis B (HBV) infection (16.21%). Only six patients had Child–Pugh B class 7 points with preserved liver function. All 37 HCC patients received curative treatment: 9 (24.32%) underwent surgical resection and 28 (75.67%) received percutaneous ultrasound-guided tumor ablation. The mean duration of follow-up was 29.23 ± 10.74 months. Within the observation time, 13 (35.13%) patients died (group HCd), while 24 survived (group HCs). One patient from the cirrhotic control group died, but without liver-related causes. Regarding tumor relapse, 22 patients (59.45%) were diagnosed with tumor recurrence.

Regarding BCLC class, the HCC patients are classified as follows: 4 BCLC 0, 15 BCLC A, and 18 BCLC B with compensated underlying liver disease. BCLC-B class is characterized by extensive heterogeneity due to the wide range of liver function (Child–Pugh A or B cirrhosis) and variable lesion number and size. Bolondi et al. proposed a sub-classification of intermediate stage HCC so that BCLC B patients might benefit from other treatment options besides transarterial chemoembolization [19]. All 18 BCLC B patients from our study had undergone curative treatment. Of these, 16 correspond to “up to seven criteria” as follows: 9 patients had tumors less than 6.5 cm diameter and underwent surgery, 7 patients had two or three nodules, with a total sum diameter less than 8 cm. The last 2 patients had four nodules, in which surgery and ablation were combined. Unfortunately, liver transplantation is not feasible in our center, so surgical resection or percutaneous ablation were also performed in patients who met the Milan criteria.

None of the patients with HCC and HCV received interferon-free treatment before diagnosis of liver tumors. One had a history of treatment with ribavirin and interferon with no virological response. Subsequently, after the curative treatment, out of the sixteen patients with HCC and HCV, eight underwent DAA treatment with sofosbuvir/ledipasvir. Of these, two had recurrence. Of the six patients with HCC and HBV, one was on lamivudine therapy. Subsequently, none of these were treated with entecavir after curative treatment. All the patients included in this study, including those with HCC and ethanolic cirrhosis, were abstinent.

The baseline characteristics of the study population are depicted in Table 1:

### 3.2. Univariate and Multivariate Analysis of Metabolic Profile

#### 3.2.1. Analysis of Raw Data Based on UHPLC-QTOF-ESI+-MS Peak Intensities 

The data released from the UHPLC-QTOF-ESI+-MS analysis were included in matrices representing the m/z values and peak intensities for each of the more than 300 molecules separated. After eliminating small signals with S/N values < 10 and molecules with peak intensities less than 25,000, the number of peaks remained at around 250. Only metabolites detected in more than 80% of the samples (*n* = 154) were subsequently included in the statistical analysis. The aligned matrix was converted to a .csv file and processed using the online software Metaboanalyst 5.0. 

Table S1 (Supplementary File) includes a list of molecules separated and identified (*n* = 154), and their average LC-MS peak intensities and standard deviations (SD). The m/z values represent [M + 1] where M is the individual molecular mass. Table S2 (Supplementary File) includes a list of the same molecules and their average intensities for the subgroups HCd (*n* = 13) vs. HCs (*n* = 24).

The main classes of molecules are represented by fatty acid derivatives (31), glycerophospholipids and lysoderivates (15 and 27, respectively), diacyl- and monoacyl glycerols and phosphoglycerols (18, 6, and 5 respectively), ceramides and sphingosine derivatives (18), sterols and bile acids (11), and acylcarnitines (11), as well amino acids, choline derivatives (9) and oxylipins as eicosanoid inflammatory mediators (4). Their relative levels in the HCC (HC group) vs. cirrhosis group (C), and their values in the HCd group vs. survivals of the HC group, are presented in Table 2.

#### 3.2.2. Discrimination Analysis: PCA and PLSDA for HC vs. C Groups

**Figure 1:**
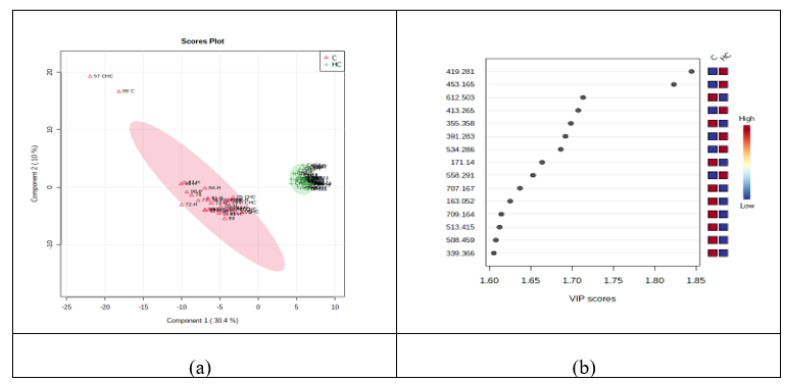
(**a**) PLSDA plot with samples’ identification, showing the discrimination between C and HC groups. (**b**) VIP scores derived from PLSDA loadings.

First, the unsupervised PCA was conducted, showing an explained co-variance of 40.6% for the first two components in the HC vs. C groups (data not shown). The discrimination between the HC and C groups was better represented by PLSDA (with a co-variance of 40.1%) (Figure 1a) and the VIP scores derived from the PLSDA loadings showed the first 15 molecules to be considered as putative biomarkers of discrimination between the two groups (Figure 1b). The cross-validation algorithm showed high accuracy (close to one), a high R2 and significantly high Q2 values > 0.95. These data indicated good predictability for this model. 

Based on the PLSDA loadings graphic and VIP scores, there were noticeably increased levels of six molecules in the HC group: 1,25-dihydroxy cholesterol (m/z = 419.281), myristyl palmitate (m/z = 453.165), 25-hydroxy vitamin D2 (m/z = 413.265), 12-ketodeoxycholic acid (m/z = 391.283), lysoPC (21:4) (m/z = 558.291), and lysoPE (22:2) (m/z = 534.286). Meanwhile, decreases in the HC group were observed for ceramide (d18:0/20:0 (2OH)) (m/z = 612.503), MG (0:0/18:2/0:0) (m/z = 355.358), decenoic acid C10 (m/z = 171.140), hydroxy adipic acid (m/z = 163.052), PG (O-16:0/16:0) (m/z = 709.164), DG (12:0/16:0/0:0) (m/z = 513.415), lysoPE (20:1) (m/z = 508.459), and dimethyl-2-eicosenoic acid (m/z = 339.366). 

Table 3 represents the data released from the *t*-test and fold-change analysis, showing the tendency of molecules to evolve between HC and C groups.

#### 3.2.3. Random Forest Analysis, Heatmaps and Biomarker Analysis for HC vs. C Groups

**Figure 2:**
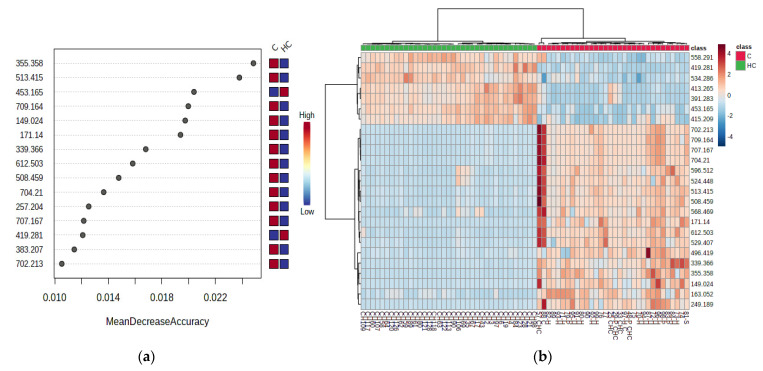
(**a**) Random forest graph showing the mean decrease in accuracy for the molecules as putative biomarkers of differentiation between the HC and C groups. (**b**) Heatmap showing the clusters and molecules responsible for the differentiation between the HC and C group.

Figure 2a,b represent the graphics of random forest analysis, and heatmaps showing the illustration of differences between the samples and groups.

**Figure 3:**
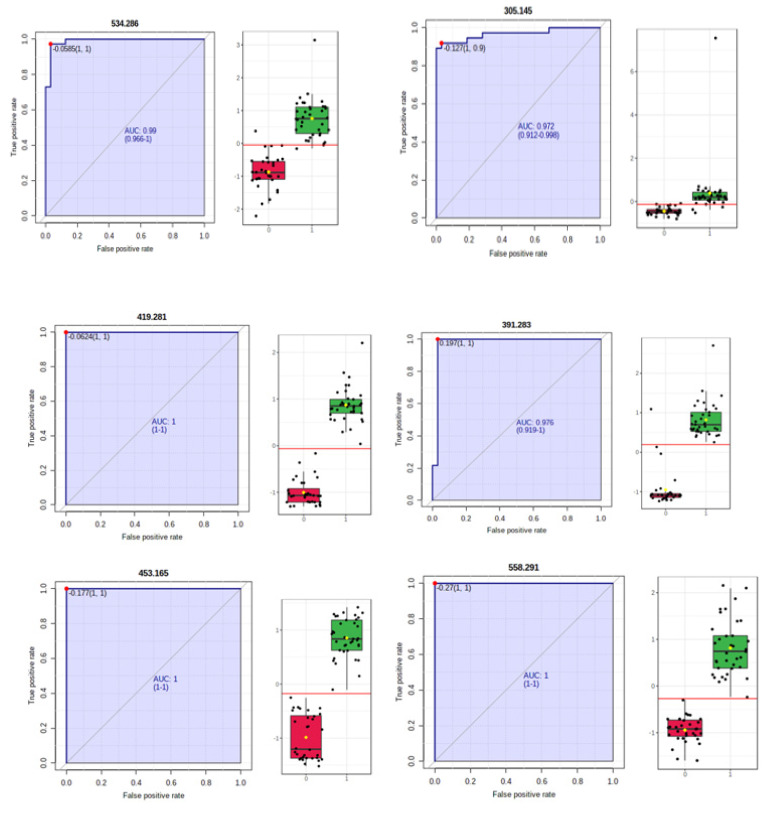
ROC curves of the most significant metabolites: 1,25-dihydroxy cholesterol (m/z = 419.281), 12-ketodeoxycholic acid (m/z = 391.283), myristyl palmitate (m/z = 453.165), lysoPC (21:4) (m/z = 558.291), lysoPE (22:2) (m/z = 534.286), and arachidonic acid (m/z = 305.145).

Biomarker analysis allowed the calculation of sensitivity versus specificity for each molecule, represented by the AUC values in the ROC curves. Table 4 shows the m/z values and molecule identifications, the AUC values higher than 0.989, the Log2FC and *p*-values of each molecule considered as a potential biomarker, and their variation in the HC vs. C groups. Simultaneously, Figure 3 illustrates the AUROC values for the six most representative metabolites in our study.

#### 3.2.4. Correlations between Lipid Metabolites, and Clinical and Biological Characteristics

The relation of different lipid metabolites and clinical and biological parameters was assessed using Spearman correlations. For each HCC patient referred for surgery, portal hypertension was measured by hepatic venous portal flow gradient (HVPG). Moreover, it has been established that values above 10 mmHg, equivalent to clinically significant portal hypertension, are associated with post hepatectomy liver failure [19]. Since HVPG measurement is not widely available and is considered an invasive procedure, the pursuit of finding biomarkers to identify patients at risk is paramount. Using Spearman correlations, we have found out that, in HCC patients who underwent surgery, C16 sphingosine tends to predict clinically significant portal hypertension (*p* = 0.06, R = −0.05), and sphingosine-1 phosphate correlates with HVPG. 

A total of 22 out of 37 HCC patients (59.45%) presented tumor recurrence at five year follow-up, after the initial diagnosis and treatment. Dismally, no metabolite could definitively predict tumor relapse. Since C16-sphingosine tends to correlate, and sphingosine-1 phosphate correlates, with HVPG, we can extrapolate and attest that C16-sphingosine and sphingosine-1 phosphate are associated with recurrence and post-hepatectomy liver failure. More mathematical models are needed which include metabolites to determine the patients at risk.

We have pointed out that 1,25-dihydroxy cholesterol (m/z = 419.281), myristyl palmitate (m/z = 453.165), 12-ketodeoxycholic acid (m/z = 391.283), lysoPC (21:4) (m/z = 558.291), and lysoPE (22:2) (m/z = 534.286) are putative biomarkers that differentiate HCC from cirrhosis. Along these lines, we have investigated whether these biomarkers have a role in decompensation and prognosis. The deterioration of liver function in a patient with cirrhosis is characterized by jaundice, ascites, hepatic encephalopathy, hepatorenal syndrome, or variceal bleeding. In total, among the included patients, eight developed ascites, three developed variceal bleeding, and one hepatic encephalopathy. In a univariate analysis among the HVPG (OR = 0.82, 95% CI: 0.8–1.24, *p* < 0.005) and MELD score (OR = 0.74, 95% CI: 0.54–0.93), arachidonic acid was the only metabolite to predict decompensation (OR = 0.1, 95% CI: 0–0.16, *p* < 0.005). In the multivariate analysis, unfortunately, no variable was associated with decompensation.

#### 3.2.5. Univariate Analysis to Predict the Death (Comparison of HCd vs. HCs Groups)

To identify which patients were at risk, Cox regression analysis was applied to further evaluate the predictive capacity of critical metabolites and assess the overall survival of HCC patients. During a follow-up period of five years, 13 (35.13%) out of 37 patients died, and, among them, 8 had developed a decompensation episode. The survival of patients with all lipid parameters as single variables was examined, using the Cox proportional hazard method for death. The highest HR for death was found for myristyl palmitate (HR = 16.67), *p* = 0.005 (Table 3). Furthermore, as adjusting variables in the multivariate Cox regression models, MELD and HVPG were added as predictors for survival. Adjusting for MELD score and HVPG in a multivariate analysis (Table 5) we have found that arachidonic acid (HR = 12.08, 95% CI: 2.95–49.93, *p* < 0.005), 1,25-dihydroxy cholesterol (HR = 10.61, 95% CI: 1.37–82.3, *p* < 0.005), myristyl palmitate (HR = 31.63, 95% CI: 1.51–661.66, *p* < 0.005), 12-keto deoxycholic acid (HR = 21.98, 95% CI: 1.39–121.39, *p* < 0.005), lysoPC (21:4) (HR = 10.46, 95% CI: 1.41–77.54, *p* < 0.005), and lysoPE (22:2) (HR = 2.37, 95% CI:1.07–5.24, *p* < 0.005), are independent markers of survival. 

#### 3.2.6. Multivariate Analysis for HCd vs. HC Groups

**Figure 4:**
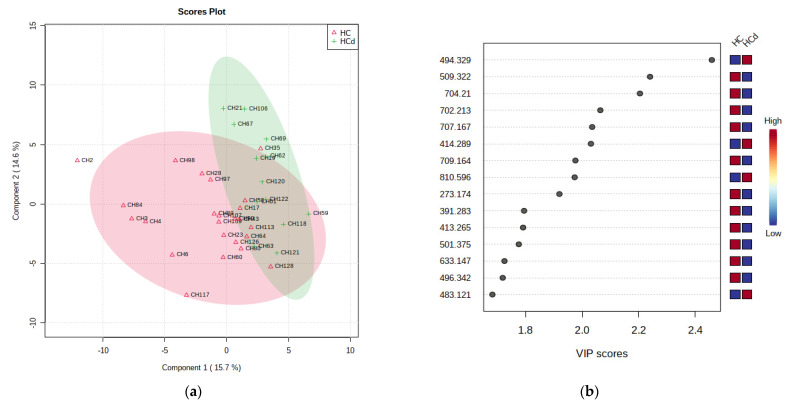
(**a**) PLSDA plot with samples’ identification, showing the discrimination between the HCd and HCs groups. (**b**) VIP scores derived from PLSDA loadings.

Figure 4 shows the PLSDA plot, which reflects the discrimination between HCd and HCs (marked HC) groups (a), and the molecules with higher VIP scores derived from PLSDA loadings.

**Figure 5:**
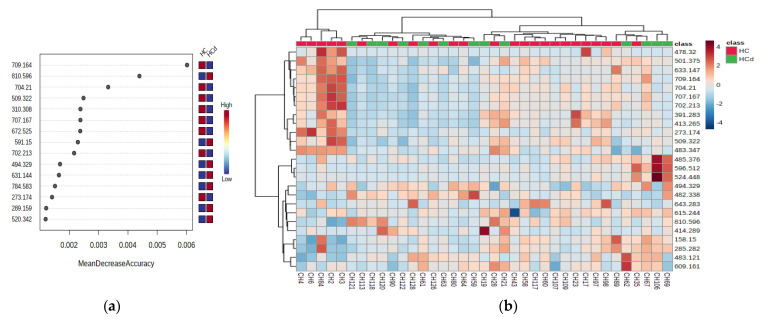
(**a**) Random forest graph showing the mean decrease in accuracy for the molecules as putative biomarkers of differentiation between the HCd and HCs groups. (**b**) Heatmap showing the clusters and molecules responsible for the differentiation between the HCd and HCs groups.

Figure 5 represents the graphics of random forest analysis (a), and heatmaps showing the illustration of differences between the groups HCd and HCs.

Table 6 shows the first 20 molecules with VIP scores higher than 1.5 and MDA values higher than 0.01, and the AUC values. 

The data obtained highlights that arachidonic acid, 1,25-dihydroxy cholesterol myristyl palmitate, 12-ketodeoxycholic acid, lysoPC (21:4), and lysoPE (22:2) may predict HCC mortality and recurrence, and constitute important biomarkers. Moreover, 12-ketodeoxycholic acid levels seem to decrease for HCC patients at risk of death, and thus might represent an important parameter.

## 4. Discussion

The early detection of hepatocellular carcinoma and the identification of prognostic factors are key to personalized medicine to improve patient outcomes. Along these lines, the main objective of our study was to identify new biomarkers for diagnosing early hepatocellular carcinoma and to identify which patients are at risk of developing tumor recurrence, or even death. Therefore, we conducted an untargeted metabolomics study emphasizing lipid metabolites in 69 European patients—32 compensated cirrhotic patients without HCC (controls) and 37 compensated cirrhotic patients with early HCC (cases) that underwent curative treatment (surgery or ablation), and have followed up with these patients for almost four years. 

The AFP values did not show any differences between HCC and cirrhosis patients, most likely due to both groups’ compensated underlying liver disease. Therefore, AFP is an imperfect surveillance tool making it imperative for the hepatology and oncology community to find a reasonably suitable hepatic cancer biomarker. Ultra-high-performance liquid chromatography coupled with electrospray ionization quadrupole time-of-flight mass spectrometry (UHPLC-ESI+-QTOF-MS) revealed important serum biomarkers that differentiate HCC patients from cirrhotic controls. The present study has identified different lipid molecules involved in fatty acid, glycerophospholipid, sphingolipid, and acylglycerol metabolism as putative biomarkers for differentiating cirrhosis and HCC, with AUC values over 0.900. Moreover, for the first time, we have identified metabolites that differentiate the deceased HCC patient from those who survived. Hence, we report two types of phosphocholines (PC (30:2); PC (30:1)) with AUROCs of 0.820 and 0.807, respectively, that are increased in early HCC, with levels that drop when tumors progress. Indeed, several studies have reported changes in gene expression and enzyme activity that led to altered PC synthesis in cancer, which contributes to tumorigenesis, while other studies have revealed oscillating PC expression in HCC patients [20,21]. To our knowledge, the present study contrasts and compares the expression of PC metabolites for the first time between early HCC patients who survived curative treatment and those who died. An emphasis on PC metabolism in the future might bring new treatment options in oncology.

Based on the PLSDA loadings graphics and VIP scores, our study identified several metabolites increased in the HCC group compared to the cirrhotic controls, depicted in Figure 5. The lipid metabolites with the most increased expression were attributed to 1,25-dihydroxy cholesterol, 25-hydroxy vitamin D, myristyl palmitate, 12-ketodeoxycholic acid, lysoPC (21:4), and lysoPE (22:2). Cholesterol plays an intricate role in liver tumorigenesis and supports the growth of hepatocarcinoma lesions depleted of fatty acid synthase in mice and humans [22]. Moreover, it seems that HMG-CoA reductase, a cornerstone enzyme of cholesterol synthesis and the target of statins, is upregulated in human HCC samples and retrospective studies suggest that the use of statins might be associated with a reduced risk of HCC development [23]. Our study found out that 1,25-dihydroxy cholesterol is upregulated by almost six times relative to cirrhotic controls.

Progressing into the lipid maze, the expression of 25-hydroxy vitamin D is a matter of debate. Undoubtedly, low levels of vitamin D are associated with increased mortality in chronic liver disease. In contrast, its increased expression in HCC serum in some studies has been demonstrated to have an antitumoral role, and, in other studies, was associated with tumor progression [24,25,26]. Bile acids, phosphatidyl-choline, and phosphatidyl-ethanolamine metabolites also play a dual role in cancer biology [17,20,27]. Although our study determines minuscule pieces from the puzzle of lipid biology, 1,25-dihydroxy cholesterol, myristyl palmitate, 12-keto deoxycholic acid, lysoPC (21:4), and lysoPE (22:2) represent important independent markers of survival in multivariate analysis, when adjusting for MELD score and portosystemic gradient. Thus, the next step for our study would be to determine a mathematical model encompassing the metabolites mentioned above, and to apply it to other cohorts of cirrhosis and liver cancer to determine which patients are at risk.

**Figure 6:**
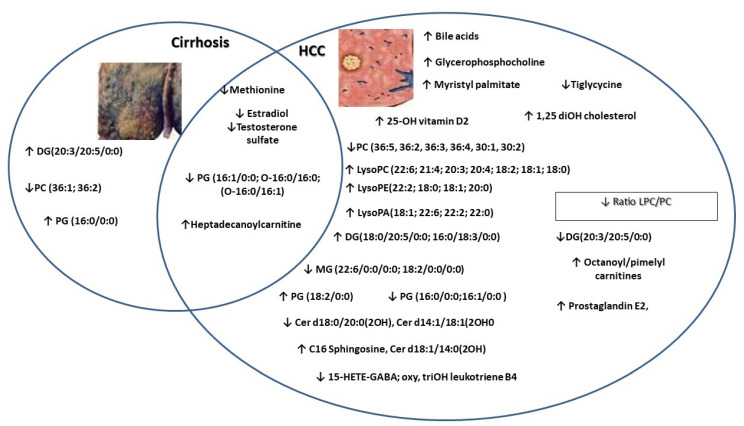
Common and specific molecules which were found to discriminate between compensated cirrhosis versus early hepatocellular carcinoma.

Figure 6 represents the general variations in specific molecules between cirrhosis versus early hepatocellular carcinoma.

In our study, we have revealed that most ceramides are expressed at lower levels compared to cirrhotic patients. This is most likely due to a depletion of ceramide content during transformation into bioactive molecules with protumor roles. Takashima, Y. and Li, Z. have confirmed these arguments and state that ceramide metabolism is deregulated in primary liver cancer [28,29]. Nonetheless, we have conceded that sphingolipid family members discriminate between HCC and cirrhosis, and might represent excellent biomarkers far better than AFP. 

Apart from being an essential player in stimulating the apoptosis of tumor cells, chemotherapy and ionizing radiation exert their effects through ceramides, a fact demonstrated by the increased levels of ceramides after exposure to those procedures [30]. Therefore, ceramides constitute crucial cancer treatment targets or adjuvant therapies to existing chemotherapies [31]. Since we have obtained increased levels of many ceramides in cirrhosis and decreased levels in HCC patients, we believe that, in future, it would be a cornerstone to determine the cut-off values of ceramides to pinpoint the high-risk moment of developing primary liver cancer. Along these lines, large cohort studies are needed which focus on this direction. Studies attest that S1P is increased in the serum of HCC patients [13]. There is an S1P axis, which refers to all the molecules involved in its metabolism and its receptors or other intracellular targets. S1P is produced (inside the cell) by the phosphorylation of sphingosine, a reaction catalyzed by two sphingosine kinases: SPHK1 and SPHK2. SPHK1 is of great importance between these two kinases, because its increased activity stimulates cell growth and inhibits apoptosis. Once activated inside the cell, S1P can act on intracellular targets or be secreted in the interstitial area and interact with cell surface receptors, a process known as “inside-out” signaling. The importance of this axis lies in the fact that its components can be therapeutic targets in cancer treatment. For example, monoclonal antibodies directed against S1P have been synthesized (sonepcizumab), alongside SPHK1 inhibitors, and various agonists and antagonists of the S1P receptors (S1PRs), and are in several phases of clinical trials [14]. Although we obtained results in contrast with those in the literature, we consider that the low level of S1P obtained in our patients with HCC is due to the presence of compensated underlying liver disease. It is likely that S1P increases in more advanced stages of cirrhosis or tumor progression. 

There is scarce data regarding the role of lipids as prognostic markers in HCC. One study from 2019 from a French group revealed elevated concentrations of phosphatidylcholine (PC) 16:0/16:1 (*p* = 0.0180), PC 16:0/16:0 (*p* = 0.0327), PC 16:0/18:1 (*p* = 0.0264) and sphingomyelin (SM) 18:2/24:1 (*p* = 0.0379), and low concentrations of lyso-phosphatidylcholine 20:4 (0.0093) and plasmalogen-phosphatidyl-ethanolamine (LysoPE) 16:0/20:4 (*p* = 0.0463), LysoPE 18:0/20:4 (*p* = 0.0077), LysoPE 18:0/20:5 (*p* = 0.0163), and LysoPE 18:0/20:3 (*p* = 0.0463) as good biomarkers for HCC versus cirrhosis, as well as two ceramides (ceramide d18:1/26:0 and ceramide d18:1/24:1) that were associated with the risk of death in one and/or three years [20]. Moreover, in a Chinese cohort, it seems that changes in polyunsaturated-eicosapentaenoic acid, docosahexaenoic acid, and linolenic acid are associated with early tumor recurrence after hepatectomy. In addition, the researchers also found that 85% of early recurrent HCCs can be predicted with an AUROC equal to 0.95 in a training set with the combination of methionine, GCDCA, and cholesterol sulfate [32]. Another Chinese study identified, after univariate and multivariate Cox regression, the combined retinol and retinal panel as an independent predictor for HCC, and showed that the low expression of the panel was correlated with decreased survival after hepatectomy. Retinol and retinal discriminate HCC from cirrhosis with an AUROC of 0.996 and 0.994 in tissue, and 0.812 and 0.744 in serum, respectively [33]. No metabolite identified in our study could determine tumor relapse. Nevertheless, since C16-sphinganine tends to correlate, and sphingosine-1 phosphate correlates, with HVPG, we can extrapolate and attest that C16-sphinganine and sphingosine-1 phosphate are associated with recurrence and post-hepatectomy liver failure. Arachidonic acid was the only metabolite to predict decompensation. In the multivariate analysis, dismally, no variable was associated with decompensation. When adjusting for MELD score and HVPG in a multivariate analysis, we report for the first time that the increased expression of 1,25-dihydroxy cholesterol, myristyl palmitate, 12-keto deoxycholic acid, lysoPC (21:4), and lysoPE (22:2) are independent markers of survival. 

Although most studies emphasize serum metabolites, other authors have performed metabolomics on tissues or other biological fluids as follows. One study from China reported that the retinol metabolic signature determined from liver biopsy and serum had considerable diagnostic and prognostic value for identifying HCC patients who would benefit from prompt therapy. Other authors claim that tissue metabolomics yields a more precise biochemical information pattern when searching for tumoral energy metabolites [33].

In the last few years, the concept of prognostic scores has been reported in oncology. Different mathematical models comprising biochemical analyses were released to assess tumor relapse or identify patients at risk of developing complications. Naturally, metabolomics also gained terrain. In 2020, Wang Q et al. reported a global prognostic index (GPI) score for operated HCC patients that combines a metabolite panel with satellite nodes for assessing overall survival. Compared with the current clinical classification systems, including the Barcelona clinic liver cancer (BCLC) stage, the tumor node metastasis (TNM) stage, and the albumin-bilirubin (ALBI) grade, the GPI score presented a notable performance according to the time-dependent receiver operating, and might stand as a helpful tool to stratify the HCC prognostic risk after surgery [34]. Other scientists used gas chromatography-mass spectrometry (GC-MS)-based metabolomics, identifying phenylalanine and galactose and including them in two mathematical models to predict the risk of mortality, recurrence, and metastasis with essential results. Moreover, some reports attest to different discriminatory biomarkers between HCC and cirrhosis when performing metabolomics protocols from urine or feces [35,36]. 

Our study presents several limitations. The number of patients was too small to conduct a complete characterization of biomarkers. These results need to be confirmed in a more significant number of patients. Additionally, more studies with European cohorts need to be performed, as we have compared our results with Chinese patients and there may be differences. Another limitation of our study is the inclusion of HCC patients with different etiologies of the underlying liver disease. Although the underlying etiology in most cases is chronic viral hepatitis C as in the control group, patients with other etiologies have been added to include a sufficient number of patients. One final limitation of our study is the inclusion of 18 patients at the BCLC B stage. Although these patients are classified as at an intermediate stage, they have a preserved liver function and have undergone curative treatments, such as surgery and percutaneous ablation.

Overall, while many metabolites appear to display excellent diagnostic performances in HCC, reproducibility stands as a significant issue to address. In the future, it will be paramount to include patients with decompensated cirrhosis, and intermediary and advanced HCC to broaden the metabolite spectrum of liver disease. In addition, in the literature, we have seen that validation cohorts have a minimal number of studies, most of them retrospective and with no statistical adjustments for important confounding variables such as smoking status, alcohol consumption, lifestyle habits, physical activity, body mass index, or waist circumference. With this in mind, given the rise of HCC developed on non-alcoholic fatty liver disease, it would be far-reaching to conduct a different study assessing this matter. Lipid metabolism is the metabolic hub of NAFLD with bioactive sphingolipids, as a hallmark of NAFLD and NAFLD-derived HCC [37]. 

## 5. Conclusions

Due to the high incidence of cancer, and particularly hepatocellular carcinoma, the search for non-invasive biomarkers is crucial in modern medicine. Such markers should be useful in the very early diagnosis of the disease, and in determining the prognosis and monitoring of the disease course and treatment. Moreover, it would be interesting to perform a metabolomic study on patients with hepatic cancer that underwent ablation, trans-arterial chemoembolization, or required systemic treatment to find out which are the best candidates for the treatment, and to monitor the patients appropriately.

In conclusion, our study reveals missing pieces of the lipid puzzle that not only differentiate compensated cirrhosis and early hepatocellular carcinoma, but also identify patients at risk. Specifically, it was determined for the first time that arachidonic acid, myristyl-palmitate, and the family members of phosphatidyl-choline are markers of survival, and might constitute future prognostic biomarkers in a personalized and minimally invasive medicine practice. We believe that by navigating the old maze of lipids discovered years ago, we could find answers to current struggles in hepatology and oncology.

## Figures and Tables

**Table 1:** Baseline characteristics of the study population.

	HCC (Group HC)	Cirrhosis (Group C)	*p* Value
**General Data**			
Number	37	32	
Age mean SD *	64.62 (6.29)	55.97 (8.1)	<0.001
Sex, no (%) **			
Females	10 (27.03)	20 (62.5)	
Males	27 (72.97)	11 (37.5)	<0.003
Etiology (%) **			
HCV	16 (43.24)	32 (100%)	
HVB	6 (16.21)		
Alcohol	12 (32.4)		
Cholestatic	1 (2.7)		
NAFLD	2 (5.4)		<0.01
Dead (yes), no (%)	13 (35.13)	1 (3.33)	<0.001
**Laboratory values**			
ALAT *	37 (21–59)	74.5 (46.25–108.25)	0.015
ASAT *	57 (35–82)	72.5 (52.25–97.75)	0.76
γGT *	61 (33.5–91.5)	60 (42–129)	0.67
Total bilirubin *	1 (0.8–1.6)	1.15 (0.9–1.35)	0.67
Creatinine *	0.73 (0.61–0.95)	0.72 (0.58–0.84)	0.2
Albumin *	3.8 (3.6–4.1)	4.1 (4–4.3)	<0.01
Hemoglobin *	14.2 (12.2–15.2)	14.5 (13.7–15.8)	0.06
PMN count *	3.37 (2.5–4.62)	3.11 (2.52–3.95)	0.089
Platelet count*	117 (82–147)	103 (79–144.5)	0.725
Na *	140 (138–142)	140 (139–143.25)	0.3
K *	4.3 (4.07–4.6)	3.9 (3.8–4.3)	0.012
AFP *	10.1 (5.5–58.8)	13.5 (8.33–19.62)	0.016
**The severity of Liver Disease**			
Child–Pugh **			
A	31 (74.1%)	32 (100%)	<0.01
B	6 (22.2%)		
MELD **			
≤9	16 (43.24%)	19 (59.38%)	0.23
10–19	1 (2.7%)	13 (40.62 %)	
	20 (54.5%)		
Portal pressure *			
HVPG	15 (11–18)	14 (11–16)	0.448
**HCC staging**			
BCLC **			
**0**	**4 (11%)**		
**A**	**15 (40%)**		
**B**	**18 (49%)**		
Tumor number **			
<3	34 (91.89%)		
3–5	3 (8.11%)		
>5	0		
Milan criteria **			
In	19 (51.35%)		
Out	18 (48.65%)		

* = values expressed as median IQR; ** = values expressed as absolute value + per cents; AFP = alpha-feto protein; HCC = hepatocellular carcinoma; *p* = level of significance; HCV = hepatitis C virus; HBV = hepatitis B virus; NAFLD = non-alcoholic fatty liver disease; ALAT = alanine aminotransferase; ASAT = aspartate aminotransferase; Γgt = gamma glutamyl transferase; Na = sodium; K = potassium; MELD = model for end stage liver disease; HVPG = hepatic venous pressure gradient; BCLC = Barcelona clinic liver cancer.

**Table 2:** Metabolites separated and identified in different patient groups (HC, C, HCd, and HCs) and the ratios of their levels in the groups HC vs. C, and HCd vs. HCs.

m/z		HC/C	HCd/HCs	m/z		HC/C	HCd/HCs
Steroids, Incl.Bile Acids				Fatty Acid Derivatives			
419.281	1,25 DiOH cholesterol	6.90	0.96	453.165	Myristyl palmitate	4.12	0.97
391.283	12-Ketodeoxycholic acid	3.24	0.89	313.255	Icosanoic (arahidic) acid C20:0	2.25	0.93
413.265	25-hydroxy vitamin D2	3.06	0.89	331.284	Docosapentenoic acid (C22:5)	2.07	0.95
409.328	Ursocholic acid	1.38	0.9	303.181	Eicosapentenoic acid (C20:5)	1.85	0.86
473.327	3-Sulfodeoxycholic acid	1.36	0.91	425.130	Lauryl palmitate	1.83	1.09
271.264	Estrone	1.17	1.13	325.250	Methyl-7-eicosenoic acid	1.79	0.92
585.268	Cholic acid glucuronide	1.14	1.19	318.291	N-methyl arachidonoyl amine	1.76	1.07
289.159	Testosterone/DHEA	1.14	1.02	397.268	Hexacosanoic acid (C26:0)	1.59	1.02
421.338	Dihomodeoxycholic acid	0.60	0.92	427.390	N-stearoyl arginine	1.23	1.03
369.295	Testosterone sulfate/DHEAS	0.55	0.92	326.354	Oleoyl Ethanolamide	1.17	1.03
273.174	Estradiol	0.50	0.7	350.338	Dihomo-gamma-linolenoyl (C18:3) Ethanolamine	1.10	1.01
**Oxylipins**				299.126	2-hydroxy oleic acid (C18:1)	1.06	1.04
353.271	Prostaglandin E2	2.54	0.93	338.341	Docosenamide (C22)	1.02	1.01
267.266	Tetranor 12-HETE	1.15	0.96	324.325	Linoleoyl ethanolamide	1.01	0.95
406.328	15-HETE-GABA	0.49	0.77	341.298	Docosanoic acid (C22:0)	0.94	0.96
383.207	12-Oxo-trihydroxy-leukotriene B4	0.22	1.06	348.319	O-Arachidonoyl (C20:4, n-6) Ethanolamine	0.86	1.06
**Acyl carnitines**				269.217	Heptadecenoic acid (C17:1)	0.65	1.03
414.289	Heptadecanoyl carnitine	3.92	2.47	501.375	Palmitoleyl linolenate	0.55	0.93
290.261	Adipoyl carnitine	2.81	1.09	301.142	Eicosahexaenoic acid (C20:6)	0.45	0.96
316.317	Decanoylcarnitine	1.90	1.1	529.407	Linoleyl linoleate	0.43	1.04
304.295	Pimelyl carnitine	1.78	1.03	285.282	Stearic acid	0.42	1.06
288.266	Octanoyl carnitine	1.66	1.08	295.182	2-Hydroxy linolenic acid (C16:3)	0.40	0.97
230.24	Butenyl carnitine	1.28	1.01	279.160	Linolenic acid (C18:3)	0.33	0.98
332.327	3-hydroxydecanoyl carnitine	1.21	0.99	163.052	Hydroxy adipic acid	0.26	1.18
312.326	Decadienoyl carnitine	1.18	1.01	249.189	Hexadecatetraenoic acid C16:4	0.22	1.05
374.259	Dodecanedioylcarnitine	1.15	0.93	229.131	Myristic acid (C14)	0.21	1.03
388.355	3-Hydroxytetradecanoyl carnitine	1.09	1.04	257.204	Palmitic acid (C16:0)	0.20	0.93
310.308	Decatrienoyl carnitine	0.98	0.93	339.366	Dimethyl-2-eicosenoic acid (C22)	0.16	1.09
**Aminoacid and choline derivatives**				245.077	Hydroxy myristic acid (C14)	0.14	1.02
258.265	Glycerophosphocholine	1.69	1.02	305.145	Arahidonic acid (C20:4)	2.83	1.51
166.073	Phenyl alanine	1.10	0.94	415.209	Ascorbyl palmitate	2.94	0.99
183.082	Phosphoryl choline	0.46	0.98	**Sphingolipids**			
161.1	Tryptamine	0.43	0.59	274.265	C16-Sphingosine	1.64	1.07
149.024	Methionine	0.27	0.98	526.518	Ceramide (d18:1/14:0 (2OH))	1.49	1.05
158.15	Tiglylglycine	0.12	1.08	554.547	Ceramide (d18:1/16:0 (2OH))	1.10	1.04
171.14	Glyceraldehyde 3-phosphate	0.06	1.04	354.360	C16 Sphinganine 1-P	0.99	1.03
364.346	a-linolenyl choline (C18:3)	0.63	0.96	623.245	Ceramide (d18:1/22:0)	0.88	1
366.374	a-linoleyl choline (C18:2)	0.87	1.09	584.464	Ceramide (d18:0/18:0 (2OH))	0.63	0.99
**Lyso Phospholipids**				582.576	Ceramide (d18:1/18:0 (2OH))	0.61	1.08
558.291	LysoPC (21:4)	2.83	1.07	628.495	Ceramide (t18:0/20:0 (2OH))	0.58	0.98
522.357	LysoPC (18:1)	2.11	1.06	540.441	Ceramide (d18:0/16:0)	0.58	1
534.286	LysoPE (22:2)	2.08	1.09	672.525	GlycoCeramide (d18:1/14:0)	0.58	0.93
437.191	LysoPA (18:1/0:0)	1.89	0.99	703.574	Sphingomyelin 18:2/16:0	0.50	1
524.37	LysoPC (18:0)	1.68	1.02	496.419	Ceramide (d15:1/16:0)	0.49	0.97
482.338	LysoPE (18:0)	1.54	1.25	596.512	Ceramide (d20:0/18:0)	0.48	1.28
483.121	LysoPA (22:6/0:0)	1.51	1.23	508.459	Ceramide (d18:2/14:0) & isom.	0.47	1.05
510.372	LysoPE (20:0)	1.49	1.21	568.469	Ceramide (d18:0/18:0)	0.45	1.23
480.332	LysoPE (18:1)	1.42	1.18	524.448	Ceramide (d14:1/18:1 (2OH)) isomizom	0.39	1.31
520.342	LysoPC (18:2)	1.41	1.16	612.503	Ceramide (d18:0/20:0 (2OH))	0.36	1.08
495.297	LysoPA (22:0/0:0)	1.38	0.97	**Monoacyl glycerols**			
546.354	LysoPC (20:3)	1.35	1.06	379.263	MG (20:4/0:0/0:0)	1.45	0.83
491.371	LysoPA (22:2/0:0)	1.25	1.14	359.313	MG (18:0/0:0/0:0)	1.15	0.98
494.329	LysoPC (16:1)	1.22	1.27	381.304	MG (20:3/0:0/0:0)	1.03	1.02
544.341	LysoPC (20:4)	1.22	1.01	357.093	MG (18:1/0:0/0:0)	0.78	1.05
568.342	LysoPC (22:6)	1.11	1.03	403.234	MG (22:6/0:0/0:0)	0.22	0.96
502.299	LysoPE (20:4)	1.09	0.9	355.358	MG (0:0/18:2/0:0)	0.15	1.08
454.294	LysoPE (16:0)	1.08	0.97	**Diacyl glycerols**			
518.325	LysoPC (18:3)	0.98	0.98	643.283	DG (18:0/20:5/0:0)	1.96	0.59
496.342	LysoPC (16:0)	0.9	0.91	591.15	DG (16:0/18:3/0:0)	1.6	1.14
542.323	LysoPC (20:5)	0.88	1.04	609.161	DG (18:1/17:0/0:0)	1.6	1.17
508.359	LysoPE (20:1)	0.74	0.93	631.144	DG (18:4/19:0/0:0)	1.23	1.09
545.402	LysoPI (14:0/0:0)	0.63	1.02	561.403	DG (14:1/18:3/0:0)	1.07	0.93
452.392	LysoPE (16:1)	0.51	0.92	607.251	DG (18:2/17:0/0:0)	1.01	1.03
478.32	LysoPE (18:2)	0.44	0.52	617.259	DG (18:2/18:2/0:0)	0.96	1.16
480.424	Lyso PC (O-16:1/0:0)	0.43	1.42	663.457	DG (20:4/20:5/0:0)	0.91	0.99
**Glycerophospholipids**				599.247	DG (18:4/17:2/0:0)	0.85	1.07
780.553	PC (36:5) [M + H]	2.05	1.29	601.264	DG (18:3/17:2/0:0)	0.83	1.09
758.568	PC (34:2) [M + H]	1.09	1.01	615.244	DG (18:2/18:3/0:0)	0.77	1.11
816.59	PC (38:2) [M + H]	0.99	1.08	603.22	DG (18:2/17:2/0:0)	0.66	1.2
744.585	PE (36:2)	0.96	1.17	589.428	DG (16:1/18:3/0:0)	0.62	1.01
784.583	PC (36:3) [M + H]	0.95	1.06	513.415	DG (12:0/16:0/0:0)	0.56	1.04
760.582	PC (34:1) [M + H]	0.92	0.98	635.143	DG (18:2/19:0/0:0)	0.48	0.94
734.569	PC (32:0) [M + H]	0.90	1.1	633.147	DG (18:3/19:0/0:0)	0.46	0.9
810.596	PC (36:1) [M + Na]	0.83	1.41	579.294	DG (16:1/17:1/0:0)	0.37	0.94
808.582	PC (36:2) [M + Na]	0.80	1.24	665.582	DG (20:3/20:5/0:0)	0.07	1.36
633.254	PA (O-16:0/16:1)	0.79	1.08	**Phosphoglycerols**			
786.602	PC (36:2) [M + H]	0.78	1.02	509.322	PG (18:2/0:0)	1.32	0.78
806.568	PC (36:3) [M + Na]	0.76	0.93	483.347	PG (16:1/0:0)	0.88	0.81
782.564	PC (36:4)[M + H]	0.69	1.14	485.376	PG (16:0/0:0)	0.48	1.2
804.55	PC (36:4) [M + Na]	0.24	1	709.164	PG (O-16:0/16:0)	0.26	0.85
704.21	PC (30:1)	0.24	0.83	707.167	PG (O-16:0/16:1)	0.26	0.84
702.213	PC (30:2)	0.23	0.83				

**Table 3:** The m/z values and identification of molecules with the most significant differences between the HC and C groups, based on the *p* values (from *t*-tests), fold-change, and Log2FC showing the tendency of evolution in the HC vs. C groups. I—Increase; D—decrease.

m/z	Identification	*p* Value	FC	Log2FC	Tendency HC vs. C
419.281	1,25-dihydroxy cholesterol	5.94 × 10^−26^	5.98	2.5805	I
453.165	Myristyl palmitate	1.45 × 10^−24^	3.35	1.7482	I
391.283	12-keto deoxycholic acid	2.36 × 10^−18^	2.61	1.385	I
534.286	lysoPE (22:2)	3.82 × 10^−18^	2.03	1.1245	I
558.291	LysoPC (21:4)	5.62 × 10^−17^	2.17	1.1177	I
413.265	25-hydroxy vitamin D2	6.06 × 10^−19^	2.48	1.3153	I
355.358	MG (18:2/0:0/0:0)	1.34 × 10^−18^	0.12	−3.0437	D
612.503	Ceramide (d18:0/20:0 (2OH))	3.56 × 10^−19^	0.28	−1.8195	D
171.140	Decenoic acid (C10:0)	2.40 × 10^−17^	0.04	−4.4131	D
707.167	PG (O-16:0/16:1)	1.83 × 10^−16^	0.20	−2.3198	D
163.052	Hydroxy adipic acid	4.27 × 10^−16^	0.21	−2.22	D
709.164	PG (O-16:0/16:0)	8.98 × 10^−16^	0.20	−2.3214	D
513.415	PG (18:0/0:0)	1.04 × 10^−15^	0.44	−1.1718	D
508.459	Ceramide (d18:2/14:0) & isom	1.42 × 10^−15^	0.37	−1.4338	D
339.366	Dimethyl eicosanoic acid (C20:1)	1.67 × 10^−15^	0.13	−2.8875	D
415.209	Ascorbyl palmitate	3.74 × 10^−13^	2.50	1.3232	D
702.213	PC (30:2)	1.31 × 10^−14^	0.17	−2.5291	D
704.210	PC (30:1)	6.58 × 10^−15^	0.18	−2.4645	D
596.512	Cer (d18:0/20:0) and isom	2.41 × 10^−13^	0.37	−1.3964	D
524.448	Ceramide (d18:1/15:0)	7.40 × 10^−13^	0.30	−1.6999	D
568.459	Ceramide (d18:0/18:0)	1.37 × 10^−12^	0.35	−1.4968	D
529.407	Linoleyl linoleate & isomers	1.72 × 10^−14^	0.33	−1.5771	D
496.419	Ceramide (d15:1/16:0)	1.04 × 10^−12^	0.41	−1.2777	D
149.024	Methionine	2.63 × 10^−13^	0.21	−2.2327	D
249.189	C16:4 fatty acid	2.46 × 10^−15^	0.18	−2.4734	D
257.204	Palmitic acid C16:0	4.62 × 10^−12^	0.15	−2.7155	D
383.207	16,16-dimethyl-PGE1	4.46 × 10^−4^	0.15	−2.6836	D

**Table 4:** The m/z values and molecules with an AUC >0.989, where the most significant *p*-values show the variations between the HC and C groups. The value and sign of the Log2FC score show the decrease (D) or increase (I) of molecule variation (negative values are associated with increases in molecules in the HC group, and positive values with decreases in the HC group). I—increase; D—decrease.

m/z	Identification	*p* Value (*t*-Tests)	Log2 FC	Variation (HC vs. C)
419.281	1,25 dihydroxy Cholesterol	1.5071 × 10^−33^	−2.138	I
453.165	Myristyl palmitate	1.4840 × 10^−29^	−2.041	I
558.291	LysoPC (21:4)	9.5558 × 10^−24^	−1.501	I
534.286	LysoPE (22:2)	7.8846 × 10^−18^	−1.056	I
513.415	PG (18:0/0:0)	5.1513 × 10^−28^	0.838	D
508.459	Ceramide (d18:2/14:0) & isom	1.2346 × 10^−32^	1.092	D
633.147	DG (18:3/19:0/0:0)[iso2]	5.7123 × 10^−13^	1.113	D
612.503	Ceramide (d18:0/20:0 (2OH))	2.3868 × 10^−28^	1.481	D
709.164	PG (O-16:0/16:0)	2.9093 × 10^−34^	1.953	D
707.167	PG (O-16:0/16:1)	6.0675 × 10^−40^	1.953	D
704.21	PC (30:1)	2.3366 × 10^−43^	2.079	D
702.213	PC (30:2)	2.4668 × 10^−36^	2.142	D
339.366	Dimethyl eicosanoic acid (C20:1)	9.6275 × 10^−15^	2.602	D
355.358	MG (0:0/18:2/0:0)	8.10591 × 10^−19^	2.750	D
171.14	Decenoic acid (C10:0)	5.9068 × 10^−22^	4.051	D

**Table 5:** Unadjusted hazard ratio with Cox proportional hazard method for death in lipid HCC metabolism and multivariate analysis of lipid components when adjusted to MELD and HVPG.

	Univariate Analysis			Multivariate Analysis		
Parameter	HR	95% CI	*p*	HR	95%CI	*p*
Arachidonic acid	1.56	(0.52–4.69)	0.429	12.08	(2.95–49.93)	**0.027**
1,25 dihydroxy Cholesterol	5.29	(1.86–15.08)	**0.002**	10.61	(1.37–82.3)	**0.024**
Myristyl palmitate	16.67	(2.3–120.69)	**0.005**	31.63	(1.51–661.66)	**0.026**
12-keto deoxycholic acid	4.96	(1.45–17)	**0.011**	12.98	(1.39–121.39)	**0.025**
LysoPC (21:4)	4.86	(1.81–13.01)	**0.002**	10.46	(1.41–77.54)	**0.022**
LysoPE (22:2)	1.54	(1.16–2.03)	**0.002**	2.37	(1.07–5.24)	**0.032**

MELD = model for end-stage liver disease; HVPG = hepatic venous pressure gradient; lysoPC = lysophosphatidylcholine; lysoPE = lysophosphatidylethanolamine.

**Table 6:** Molecules with VIP scores > 1.5 (see Figure 3) and MDA values > 0.01 (see Figure 4).

**m/z**	**Identification**	**AUC**	***p*-Value**	**HCd vs. HC**	**m/z**	**Identification**	**AUC**	***p*-Value**	**HCd vs. HC**
702.213	PC (30:2)	0.820	0.027	D	391.283	12-Ketodeoxycholic acid	0.714	0.064	D
704.210	PC (30:1)	0.807	0.017	D	413.265	25-hydroxy vitamin D2	0.692	0.072	D
707.167	PG (O-16:0/16:1)	0.804	0.025	D	273.174	Estradiol	0.692	0.061	D
709.164	PG (O-16:0/16:0)	0.791	0.025	D	483.121	PG (16:1/0:0)	0.684	0.064	I
509.322	PG (18:2/0:0)	0.772	0.017	D	501.375	Palmitoleyl linolenate	0.682	0.062	D
810.596	PC (36:1)	0.762	0.034	I	633.254	PA (O-16:0/16:1)	0.639	0.085	D
494.329	LysoPC (16:1)	0.761	0.017	I	591.150	DG (16:0/18:3/0:0)	0.637	0.127	I
414.289	Heptadecanoyl carnitine	0.642	0.046	I	496.342	LysoPC (16:0)	0.634	0.139	D
672.525	GlycoCeramide (d18:1/14:0)	0.631	0.160	D	631.144	DG (18:4/19:0/0:0)	0.625	0.240	I
784.583	PC (36:3)	<0.600	>0.05	I	310.308	Decatrienoyl carnitine	<0.600	>0.05	D

## Data Availability

Not applicable.

## Supplementary Materials

The following supporting information can be downloaded at: https://www.mdpi.com/article/10.3390/jcm11051292/s1, Table S1: Molecules separated and identified (n = 154) by LC-MS: average peak intensities, standard deviation (SD). The m/z values represent [M + 1] values where M- molecular mass; Table S2: Molecules separated and identified (n = 154) by LC-MS: average peak intensities (Id, I), standard deviations (SDd, SD)s for group HCd (n = 13) vs HCs (n = 24). The m/z values represent [M + 1] values where M- molecular mass.
