# Supplementary material for: Navigating through the Lipid Metabolism Maze: Diagnosis and Prognosis Metabolites of Hepatocellular Carcinoma versus Compensated Cirrhosis
Source: J Clin Med. 2022 Feb 26;11(5):1292. doi: 10.3390/jcm11051292 (PMC8910918; doi:10.3390/jcm11051292)
Supplement: Supplementary file 1 [file jcm-11-01292-s001.zip › jcm-1599867-supplementary.pdf]

Journal of
Clinical Medicine

MDPI

Supplementary Materials

Table S1. Molecules separated and identified (n=154) by LC-MS: average peak intensities, standard deviation (SD). The m/z values represent [M+1] values where M- molecular mass.

		Group C		Group HC	
**m/z**	**Identification**	**Intensity**	**SD**	**Intensity**	**SD**
149.024	Methionine	6701827	2784216	1798625	158813
158.150	Tiglylglycine	1038375	1155377	126523	15627
161.100	Tryptamine	247982	130167	107334	140276
163.052	Hydroxy adipic acid	336763	165482	88422	66643
166.073	Phenyl alanine	2328293	927027	2561620	1012768
171.140	Glyceraldehyde 3-phosphate	396098	159071	23890	2874
183.082	Phosphoryl choline	218227	350640	101061	13170
229.131	Myristic acid (C14)	343045	125408	70959	25060
230.240	Butenyl carnitine	1723064	1168536	2206002	376884
245.077	Hydroxy myristic acid (C14)	2193807	1054208	303510	39453
249.189	C16:4 fatty acid	2465657	952000	550821	57513
257.204	Palmitic acid	220699	90421	43532	11599
258.265	Glycerophosphocholine	125436	66856	211480	28435
267.266	Tetranor 12-HETE	1100228	250794	1265579	228492
269.217	Heptadecenoic acid (C17:1)	233792	297331	152228	29853
271.264	Estrone	207493	71929	241772	111308
273.174	Estradiol	433737	227717	216342	111764
274.265	C16-Sfingosine	20722582	13786721	33941132	6082164
279.160	Linolenic acid (C18:3)	3249904	1975063	1069143	105100
285.282	Stearic acid	191046	95645	81184	8346
288.266	Octanoyl carnitine	1216538	595753	2023688	506526
289.159	Testosterone/DHEA	2135674	843361	2427573	404446
290.261	Adipoyl carnitine	1918737	1569869	5386567	2270523
295.182	2-Hydroxy linolenic acid (C16:3)	201598	81886	81387	9347
299.126	2-hydroxy oleic acid (C18:1)	366971	240835	389474	134346
301.142	Eicosahexaenoic acid (C20:6)	6655506	4010548	3017794	408022
303.181	Eicosapentenoic acid (C20:5)	341160	261166	630007	259951
304.295	Pimelyl carnitine	562669	535642	1002788	136645
305.145	Arahidonic acid (C20:4)	130276	50720	368426	356592
310.308	Decatrienoyl carnitine	2259884	898886	2221515	513331
312.326	Decadienoyl carnitine	2987527	753968	3518564	778267
313.255	Icosanoic (arahidic) acid C20:0	3030917	2677145	6816297	1478805
316.317	Decanoylcarnitine	1061795	585772	2021026	446904
318.291	N-methyl arachidonoyl amine	1808172	1473867	3173960	528249
324.325	Linoleoyl ethanolamide	173720	58551	175704	52979
325.250	Methyl-7-eicosenoic acid	421342	265114	754607	152485
326.354	Oleoyl Ethanolamide	1471999	565049	1716492	456992
331.284	Docosapentenoic acid (C22:5)	2329932	1668859	4813692	820416
332.327	3-hydroxydecanoyl carnitine	727567	332116	877108	100495
338.341	Docosenamide (C22)	2860781	1376631	2914511	882634
339.366	Crease)	217275	111392	35786	6948

341.298	Docosanoic acid (C22:0)	6535286	3140999	6145643	1133764
348.319	O-Arachidonoyl (C20:4, n-6) Ethanolamine	154383	50563	133501	69751
350.338	Dihomo-gamma-linolenoyl (C18:3) Ethanolamine	184491	75939	202023	38650
353.271	Prostaglandin E2	11267880	12998099	28599745	5800758
354.360	C16 Sphinganine 1-P	477799	197555	474316	92320
355.358	MG(0:0/18:2/0:0)	163244	68779	24256	3980
357.093	MG(18:1/0:0/0:0)	58683	27773	46027	17209
359.313	MG(18:0/0:0/0:0)	4571789	1974084	5256059	836303
364.346	a-linolenyl choline (C18:3)	218571	77565	138078	60866
366.374	a-linoleyl choline (C18:2)	228632	137649	199828	72566
369.295	Testosterone sulfate/DHEAS	856910	337689	470453	72760
374.259	Dodecanedioylcarnitine	1416126	1033089	1633561	329374
379.263	MG(20:4/0:0/0:0)	295987	144575	429495	176753
381.304	MG(20:3/0:0/0:0)	35887505	12660992	37098649	4924386
383.207	12-Oxo-20-trihydroxy-leukotriene B4	810949	690817	180176	39461
388.355	3-Hydroxytetradecanoyl carnitine	137164	38252	149316	28793
391.283	12-Ketodeoxycholic acid	310348	186848	1004701	176437
397.268	Hexacosanoic acid (C26:0)	637071	159127	1015284	110376
403.234	MG(0:0/22:6)	1474289	618189	319975	88974
406.328	15-HETE-GABA	481245	159793	238073	155761
409.328	Ursocholic acid	350554	99385	484965	167065
413.265	25-hydroxy vitamin D2	985676	510680	3019905	545169
414.289	Heptadecanoyl carnitine	619889	842844	2431705	3465350
415.209	Ascorbyl palmitate	395480	338287	1160899	245520
419.281	1,25 DiOH cholesterol	176735	155411	1219696	211383
421.338	Dihomodeoxycholic acid	126467	48685	75381	11590
425.130	Lauryl palmitate	90970	34084	166221	64165
427.390	N-stearoyl arginine	1194504	488662	1468721	90740
437.191	PA(18:1/0:0)	575848	346840	1085524	162493
452.392	LysoPE (16:1)	483618	157055	247701	42584
453.165	Myristyl palmitate	202041	143278	831478	122419
454.294	LysoPE (16:0)	701857	279045	758253	186395
473.327	3-Sulfodeoxycholic acid	1300117	449374	1763374	361501
478.320	LysoPE (18:2)	604244	332742	267000	268117
480.332	LysoPE (18:1)	789578	319179	1124676	702179
480.424	Lyso PC(O-16:1/0:0)	158512	62512	68301	48182
482.338	LysoPE (18:0)	1728224	1135059	2659810	1117350
483.121	PA(22:6/0:0)	98129	64551	147874	49967
483.347	PG(16:1/0:0)	298281	113336	262861	103954
485.376	PG(16:0/0:0)	363001	122918	172853	56578
491.371	PA(22:2/0:0)	202839	142421	254550	156549
494.329	LysoPC (16:1)	1280050	724637	1567301	481384
495.297	PA(22:0/0:0)	1555734	493716	2147890	272725
496.342	LysoPC (16:0) [M+H]	52262042	18635660	46996675	8138058
496.419	Ceramide(d15:1/16:0)	558166	238512	274451	30722
501.375	Palmitoleyl linolenate	762628	174904	416632	43536

502.299	LysoPE (20:4)	767109	329725	837595	253398
508.359	LysoPE (20:1)	682051	343209	501912	496425
508.459	Cer(d18:2/14:0) and isom...	93896	12266	44027	5581
509.322	PG(18:2/0:0)	134902	34110	178153	51623
510.372	LysoPE (20:0)	1017755	588628	1517101	704811
513.415	DG(12:0/16:0/0:0)[iso2]	186296	25392	104214	8618
518.325	LysoPC(18:3)	3219452	982877	3152937	631876
520.342	LysoPC(18:2)	16702516	11083218	23547939	10047236
522.357	LysoPC(18:1)	9455344	6726416	19923598	7943606
524.370	LysoPC(18:0)	21807403	17611990	36738590	9139180
524.448	Ceramide(d14:1/18:1(2OH)) si izom	179222	41551	69724	33991
526.518	Ceramide(d18:1/14:0(2OH))	393455	209175	584550	157119
529.407	Linoleyl linoleate	259621	74479	111076	16221
534.286	LysoPE(22:2)	266344	94934	553927	107553
540.441	Cer(d18:0/16:0)	536792	203416	313308	47579
542.323	LysoPC(20:5)	1533032	644297	1355320	369107
544.341	LysoPC(20:4)	5870528	2859221	7133302	2407315
545.402	PI(14:0/0:0)	434281	136522	273359	34774
546.354	LysoPC(20:3)	4266290	2472298	5761194	1602019
554.547	Ceramide(d18:1/16:0(2OH))	555454	327700	612576	179220
558.291	LysoPC(21:4)	489929	140846	1387227	301452
561.403	DG(14:1/18:3/0:0)[iso2]	370427	174972	397565	191250
568.342	LysoPC(22:6)	1218167	881654	1350734	663025
568.469	Ceramide(d18:0/18:0)	176187	41942	79943	43891
579.294	DG(16:1/17:1/0:0)[iso2]	1954952	971875	725715	111324
582.576	Ceramide(d18:1/18:0(2OH))	226005	109073	138025	56725
584.464	Ceramide(d18:0/18:0(2OH))	395317	192615	248973	35525
585.268	Cholic acid glucuronide	849521	710096	964419	524470
589.428	DG(16:1/18:3/0:0)[iso2]	298793	116298	185907	21329
591.150	DG(16:0/18:3/0:0)[iso2]	197600	81207	316292	82467
596.512	Ceramide(d20:0/18:0)	126256	30516	60224	22594
599.247	DG(18:4/17:2/0:0)[iso2]	86871	32108	74132	20571
601.264	DG(18:3/17:2/0:0)[iso2]	229716	74253	191373	36002
603.220	DG(18:2/17:2/0:0)[iso2]	52693	45039	34627	20596
607.251	DG(18:2/17:0/0:0)[iso2]	116186	103945	117127	76860
609.161	DG(18:1/17:0/0:0)[iso2]	288706	131737	461201	130660
612.503	Ceramide(d18:0/20:0(2OH))	126142	22772	45170	12227
615.244	DG(18:2/18:3/0:0)[iso2]	51566	10470	39771	8128
617.259	DG(18:2/18:2/0:0)[iso2]	109353	51926	104842	34130
623.245	Ceramide (d18:1/22:0)	28447	6022	25055	7096
628.495	Ceramide (t18:0/20:0(2OH))	241335	97848	141124	18983
631.144	DG(18:4/19:0/0:0)[iso2]	566266	236427	695539	148771
633.147	DG(18:3/19:0/0:0)[iso2]	119678	42831	55303	9489
633.254	PA(O-16:0/16:1)	43415	11359	34192	7015
635.143	DG(18:2/19:0/0:0)[iso2]	50964	18380	24312	4376
643.283	DG(18:0/20:5/0:0)	38588	44182	75614	67919
663.457	DG(20:4/20:5/0:0)	1802171	532742	1646119	324471
665.582	DG(20:3/20:5/0:0)	609215	1262648	43125	51900

672.525	GlycoCeramide(d18:1/14:0)	134693	45784	78538	11254
702.213	PC(30:2)	243001	42259	55021	13469
703.574	Sphingomielin 18:2/16:0	3116001	2010124	1546923	512102
704.210	PC(30:1)	124667	15986	29490	6468
707.167	PG(O-16:0/16:1)	140612	19751	36294	8264
709.164	PG(O-16:0/16:0)	74405	13537	19207	3974
734.569	PC (32:0)[M+H]	317143	209475	284861	113994
744.585	PE(33:2)[M+H]	180687	126516	173541	77618
758.568	PC (34:2) [M+H]	5800325	4042261	6306546	1782385
760.582	PC (34:1) [M+H]	2993731	1620105	2744344	1206447
780.553	PC (36:5)[M+H]	1902911	1017269	3905629	2108327
782.564	PC (36:4)[M+H]	3179553	2040524	2180895	1090206
784.583	PC (36:3)[M+H]	1527850	1099340	1457082	597105
786.602	PC (36:2)[M+H]	2237847	1589170	1753216	803226
804.550	PC (36:4)[M+Na]	1460749	1094215	345705	247446
806.568	PC (36:3)[M+Na]	1241047	742771	943620	639127
808.582	PC (36:2)[M+Na]	1432347	633945	1145672	630545
810.596	PC (36:1)[M+Na]	911084	717536	757156	378335
816.590	PC (38:2)[M+H]	111039	76723	110351	71809

Table S2. Molecules separated and identified (n=154) by LC-MS: average peak intensities (Id, I), standard deviations (SDd, SD)s for group HCd (n=13) vs HCs (n=24). The m/z values represent [M+1] values where M- molecular mass.

		Group HCd		Group HCs	
**m/z**	**Identification**	**Id**	**SDd**	**I**	**SD**
149.024	Methionine	1775671	145176	1811059	167398
158.150	Tiglylglycine	133192	5423	122910	18114
161.100	Tryptamine	74234	22700	125264	171976
163.052	Hydroxy adipic acid (C6)	98277	76769	83084	61581
166.073	Phenyl alanine	2462527	1242002	2615296	889981
171.140	D-Glyceraldehyde 3-phosphate	24464	2221	23580	3173
183.082	Phosphoryl choline	99721	8564	101787	15220
229.131	Myristic acid (C14)	72549	25937	70098	25095
230.240	Butenyl carnitine	2218269	317827	2199358	411693
245.077	Hydroxy myristic acid (C14)	306549	19586	301864	47203
249.189	Hexadecatetraenoic acid C16:4	566693	28518	542224	67331
257.204	Palmitic acid (C16:0)	41457	11605	44656	11686
258.265	Glycerophosphocholine	213760	21749	210244	31847
267.266	Tetranor 12-HETE	1234699	62881	1282305	280755
269.217	Heptadecenoic acid (C17:1)	155304	39477	150562	23951
271.264	Estrone	261454	109144	231112	113311
273.174	Estradiol	169762	37197	241573	130150
274.265	C16-Sfingosine	35376417	2572128	33163687	7256364
279.160	Linolenic acid (C18:3)	1054856	89903	1076882	113554
285.282	Stearic acid (C18:0)	84430	4352	79425	9484
288.266	Octanoyl carnitine	2121963	321343	1970456	582490
289.159	Testosterone/DHEA	2461538	362878	2409175	431654
290.261	Adipoyl carnitine	5704458	2343573	5214377	2261748
295.182	2-Hydroxy linolenic acid (C18:3)	80036	5011	82118	11048

299.126	2-hydroxy oleic acid (C18:1)	398616	111258	384521	147374
301.142	Eicosahexaenoic acid (C20:6)	2936828	355670	3061650	434589
303.181	Eicosapentenoic acid (C20:5)	571880	122195	661492	308276
304.295	Pimelyl carnitine	1023127	104581	991772	152180
305.145	Arahidonic acid (C20:4)	471733	592432	312468	81312
310.308	Decatrienoyl carnitine	2125181	583347	2273696	476253
312.326	Decadienoyl carnitine	3546401	843125	3503486	759272
313.255	Icosanoic (arahidic) acid C20:0	6515661	560362	6979142	1783348
316.317	Decanoylcarnitine	2149942	362597	1951196	479095
318.291	N-methyl arachidonoyl amine	3319300	465926	3095234	552358
324.325	Linoleoyl ethanolamide	169898	54933	178850	52815
325.250	Methyl-7-eicosenoic acid	716589	151955	775200	151945
326.354	Oleoyl Ethanolamide	1749699	566307	1698505	398243
331.284	Docosapentenoic acid (C22:5)	4644454	368508	4905362	978628
332.327	3-hydroxydecanoyl carnitine	870264	94989	880815	105162
338.341	Docosenamide (C22)	2935431	755227	2903179	959899
339.366	Dimethyl-2-eicosenoic acid (C22)	37710	3741	34743	8064
341.298	Docosanoic acid (C22:0)	5990646	310389	6229599	1393114
348.319	O-Arachidonoyl (C20:4, n-6) Ethanolamine	138988	85643	130529	61335
350.338	Dihomo-gamma-linolenoyl (C18:3) Ethanolamine	203870	39005	201023	39261
353.271	Prostaglandin E2	27353661	3298593	29274707	6755574
354.360	C16 Sphinganine 1-P	484459	100246	468821	89485
355.358	MG(0:0/18:2/0:0)	25454	3218	23606	4259
357.093	MG(18:1/0:0/0:0)	47582	9614	45185	20327
359.313	MG(18:0/0:0/0:0)	5170616	261046	5302341	1026061
364.346	a-linolenyl choline (C18:3)	134574	53240	139977	65643
366.374	a-linoleyl choline (C18:2)	210791	82373	193890	67804
369.295	Testosterone sulfate/DHEAS	445694	28598	483865	85588
374.259	Dodecanedioylcarnitine	1560027	369828	1673392	306161
379.263	MG(20:4/0:0/0:0)	378244	78356	457255	208345
381.304	MG(20:3/0:0/0:0)	37511319	1164433	36875119	6090980
383.207	12-Oxo-20-trihydroxy-leukotriene B4	186863	27541	176553	44750
388.355	3-Hydroxytetradecanoyl carnitine	153552	27441	147022	29818
391.283	12-Ketodeoxycholic acid	932150	89579	1043999	199879
397.268	Hexacosanoic acid (C26:0)	1026250	42127	1009344	134306
403.234	MG(0:0/22:6)	312201	108263	324186	78886
406.328	15-HETE-GABA	200241	15143	258565	191331
409.328	Ursocholic acid	451624	42313	503025	204410
413.265	25-hydroxy vitamin D2	2801466	299116	3138225	613954
414.289	Heptadecanoyl carnitine	3961324	4985179	1603162	1947074
415.209	Ascorbyl palmitate	1149940	183230	1166835	277002
419.281	1,25 DiOH cholesterol	1186158	131365	1237862	244856
421.338	Dihomodeoxycholic acid	71604	13658	77427	10024
425.130	Lauryl palmitate	175753	50524	161058	70945
427.390	N-stearoyl arginine	1494694	56279	1454652	103185
437.191	PA(18:1/0:0)	1075810	135575	1090787	177921

452.392	LysoPE (16:1)	234370	21682	254923	49378
453.165	Myristyl palmitate	815322	101587	840229	133586
454.294	LysoPE (16:0)	741291	217833	767440	171385
473.327	3-Sulfodeoxycholic acid	1653925	531375	1822659	216311
478.320	LysoPE (18:2)	166049	33685	321682	321007
480.332	LysoPE (18:1)	1247207	846799	1058305	620109
480.424	Lyso PC(O-16:1/0:0)	84582	71916	59482	26538
482.338	LysoPE (18:0)	3059795	1433459	2443151	861769
483.121	PA(22:6/0:0)	168458	48947	136725	47840
483.347	PG(16:1/0:0)	227196	56252	282180	118975
485.376	PG(16:0/0:0)	193575	82332	161628	33158
491.371	PA(22:2/0:0)	276387	204971	242721	126586
494.329	LysoPC (16:1)	1817474	418942	1431790	465403
495.297	PA(22:0/0:0)	2099647	258720	2174021	281900
496.342	LysoPC (16:0)	44295288	11407691	48459926	5422694
496.419	Ceramide(d15:1/16:0)	269245	25582	277271	33350
501.375	Palmitoleyl linolenate	398587	30382	426406	46919
502.299	LysoPE (20:4)	779836	217464	868880	270043
508.359	LysoPE (20:1)	479996	494443	513783	507699
508.459	Cer(d18:2/14:0) and isom...	45370	6677	43299	4891
509.322	PG(18:2/0:0)	151231	31977	192735	54828
510.372	LysoPE (20:0)	1710599	970486	1412289	503501
513.415	DG(12:0/16:0/0:0)[iso2]	107042	7240	102682	9051
518.325	LysoPC(18:3)	3111186	614645	3175552	652927
520.342	LysoPC(18:2)	25851271	11866515	22300301	8939391
522.357	LysoPC(18:1)	20688808	8944849	19509109	7517436
524.370	LysoPC(18:0)	37153592	9957415	36513798	8879955
524.448	Ceramide(d14:1/18:1(2OH)) si izom	82542	51797	62781	16292
526.518	Ceramide(d18:1/14:0(2OH))	604218	147304	573896	164260
529.407	Linoleyl linoleate	113966	19207	109510	14564
534.286	LysoPE(22:2)	586070	138414	536517	84911
540.441	Cer(d18:0/16:0)	313056	39428	313445	52270
542.323	LysoPC(20:5)	1391343	307958	1335807	403278
544.341	LysoPC(20:4)	7176115	2491831	7110111	2414437
545.402	PI(14:0/0:0)	276174	32938	271834	36329
546.354	LysoPC(20:3)	5998153	2069757	5632841	1316463
554.547	Ceramide(d18:1/16:0(2OH))	629786	172889	603254	185530
558.291	LysoPC(21:4)	1449212	316475	1353653	294334
561.403	DG(14:1/18:3/0:0)[iso2]	378649	240365	407811	163692
568.342	LysoPC(22:6)	1375690	740870	1337216	633367
568.469	Ceramide(d18:0/18:0)	90938	44818	73987	43151
579.294	DG(16:1/17:1/0:0)[iso2]	696362	115976	741614	107841
582.576	Ceramide(d18:1/18:0(2OH))	144842	50855	134332	60387
584.464	Ceramide(d18:0/18:0(2OH))	247743	30919	249640	38409
585.268	Cholic acid glucuronide	1073630	479020	905264	548116
589.428	DG(16:1/18:3/0:0)[iso2]	187694	24132	184940	20135
591.150	DG(16:0/18:3/0:0)[iso2]	344499	94515	301012	72738
596.512	Ceramide(d20:0/18:0)	70165	31678	54839	13760

599.247	DG(18:4/17:2/0:0)[iso2]	77307	17602	72413	22179
601.264	DG(18:3/17:2/0:0)[iso2]	202741	19426	185216	41464
603.220	DG(18:2/17:2/0:0)[iso2]	38819	21894	32357	19964
607.251	DG(18:2/17:0/0:0)[iso2]	119019	59891	116102	85860
609.161	DG(18:1/17:0/0:0)[iso2]	508395	140734	435638	120196
612.503	Ceramide(d18:0/20:0(2OH))	47516	8748	43899	13757
615.244	DG(18:2/18:3/0:0)[iso2]	42573	3315	38254	9530
617.259	DG(18:2/18:2/0:0)[iso2]	115261	28950	99199	35936
623.245	Ceramide (d18:1/22:0)	25024	3869	25072	8426
628.495	Ceramide (t18:0/20:0(2OH))	139379	16173	142069	20614
631.144	DG(18:4/19:0/0:0)[iso2]	734983	122537	674174	159539
633.147	DG(18:3/19:0/0:0)[iso2]	51662	5522	57275	10653
633.254	PA(O-16:0/16:1)	35817	4451	33311	8024
635.143	DG(18:2/19:0/0:0)[iso2]	23353	3779	24832	4660
643.283	DG(18:0/20:5/0:0)	52202	24012	88296	80261
663.457	DG(20:4/20:5/0:0)	1636720	321949	1651210	332615
665.582	DG(20:3/20:5/0:0)	52160	62039	38231	46227
672.525	GlycoCeramide(d18:1/14:0)	74986	7390	80462	12600
702.213	PC(30:2)	48483	5824	58562	15133
703.574	Sphingomielin 18:2/16:0	1550386	527810	1545048	514896
704.210	PC(30:1)	26114	2858	31319	7162
707.167	PG(O-16:0/16:1)	32231	4078	38496	9155
709.164	PG(O-16:0/16:0)	17255	2821	20264	4153
734.569	PC (32:0)[M+H]	302454	138491	275332	100313
744.585	PE(33:2)[M+H]	191491	105308	163818	57992
758.568	PC (34:2) [M+H]	6335647	1910987	6290783	1751135
760.582	PC (34:1) [M+H]	2711618	911689	2762071	1357794
780.553	PC (36:5)[M+H]	4567709	2230672	3547002	1994835
782.564	PC (36:4)[M+H]	2375570	1288724	2075446	980207
784.583	PC (36:3)[M+H]	1508240	427058	1429372	678690
786.602	PC (36:2)[M+H]	1771380	754660	1743378	844045
804.550	PC (36:4)[M+Na]	346403	285439	345326	230928
806.568	PC (36:3)[M+Na]	900653	336979	966894	760598
808.582	PC (36:2)[M+Na]	1312198	845916	1055470	474134
810.596	PC (36:1)[M+Na]	934310	354284	661198	362226
816.590	PC (38:2)[M+H]	115844	79696	107376	68781
